# Targeting the Roots of Psychosis: The Role of Aberrant Salience

**DOI:** 10.3390/pediatric17030063

**Published:** 2025-06-04

**Authors:** Giuseppe Marano, Francesco Maria Lisci, Greta Sfratta, Ester Maria Marzo, Francesca Abate, Gianluca Boggio, Gianandrea Traversi, Osvaldo Mazza, Roberto Pola, Eleonora Gaetani, Marianna Mazza

**Affiliations:** 1 Unit of Psychiatry, Fondazione Policlinico Universitario Agostino Gemelli IRCCS, 00168 Rome, Italysfrattagreta24@gmail.com (G.S.); mariannamazza@hotmail.com (M.M.); 2 Department of Neurosciences, Università Cattolica del Sacro Cuore, 00168 Rome, Italy; 3 Unit of Medical Genetics, Department of Laboratory Medicine, Ospedale Isola Tiberina-Gemelli Isola, 00186 Rome, Italy; gianandrea.traversi@gmail.com; 4 Spine Surgery Department, Bambino Gesù Children’s Hospital IRCCS, 00168 Rome, Italy; osvaldo.mazza1973@hotmail.it; 5 Section of Internal Medicine and Thromboembolic Diseases, Department of Internal Medicine, Fondazione Policlinico Universitario Agostino Gemelli IRCCS, Università Cattolica del Sacro Cuore, 00168 Rome, Italy; roberto.pola@policlinicogemelli.it; 6 Department of Translational Medicine and Surgery, Fondazione Policlinico Universitario Agostino Gemelli IRCCS, Università Cattolica del Sacro Cuore, 00168 Rome, Italy; eleonora.gaetani@unicatt.it; 7 Unit of Internal Medicine, Cristo Re Hospital, 00167 Rome, Italy

**Keywords:** aberrant salience, psychosis, early interventions, ultra-high risk (UHR)

## Abstract

Aberrant salience, defined as the inappropriate attribution of significance to neutral stimuli, is increasingly recognized as a critical mechanism in the onset of psychotic disorders. In young individuals at ultra-high risk (UHR) for psychosis, abnormal salience processing may serve as a precursor to full-blown psychotic symptoms, contributing to distorted perceptions and the onset of psychotic ideation. This review examines current literature on aberrant salience among UHR youth, exploring its neurobiological, psychological, and behavioral dimensions. Through a comprehensive analysis of studies involving neuroimaging, cognitive assessments, and symptomatology, we assess the consistency of findings across diverse methodologies. Additionally, we evaluate factors contributing to aberrant salience, including neurochemical imbalances, dysregulation in dopamine pathways, and environmental stressors, which may jointly increase psychosis vulnerability. Identifying aberrant salience as a measurable trait in UHR populations could facilitate earlier identification and targeted interventions. Implications for clinical practice are discussed, highlighting the need for specialized therapeutic approaches that address cognitive and emotional dysregulation in salience attribution. Recent research underscores the importance of aberrant salience in early psychosis research and advocates for further studies on intervention strategies to mitigate progression to psychosis among UHR individuals.

## 1. Introduction

The term At-Risk Mental State (ARMS), also known as Clinical High Risk for Psychosis (CHR-P), first appeared in the journal Schizophrenia Bulletin two decades ago, in 1996, in a study published by Yung and colleagues [1]. In this work, the authors discussed the possibility of identifying individuals who were close to an “imminent development of a first episode of a psychotic spectrum disorder” [1]. Since then, the interest in this condition within the medical literature has grown to the point where, today, the field of mental health has embarked on an innovative, albeit challenging, journey toward a radical and substantial transformation of knowledge, treatment, and organizational structures. Preventing individuals from developing a full-blown psychotic episode—the goal of Early Psychosis Intervention centers—would have a significant impact, halting the cognitive, functional, and social disability consequences that such an event entails.

Most patients with psychotic symptoms, considered as Schneiderian first-rank symptoms (i.e., delusions, hallucinations, and thought influence experiences), come to clinical attention long after their onset [2]. This delay makes the phenomenological reconstruction of psychosis onset quite complex. Neuroscientific research, through phenomenological analyses of onset and Early Detection projects, has identified a “prodromal period” of psychosis—a process that evolves through a series of stages in which increased emotional sensitivity combines with subclinical subjective disturbances affecting affective, cognitive, verbal, somatic-perceptual, motor, and vegetative domains [3]. During this phase, individuals begin to perceive incomprehensible changes in their surroundings, prompting a need to “make sense” of their experience. This process may gradually lead to the formation of delusions [4].

The central experience of this phase may be related to aberrant salience (AS), a phenomenon where normally neutral stimuli become “salient”, meaningful, and capable of capturing attention, contributing to a sense of “revelation”. Previously incomprehensible circumstances and perceptual experiences begin to take on increasingly defined meanings. This revelatory process is often accompanied by sensations of heightened perception (“I have developed a greater awareness of…”, “I became fascinated by small things that previously seemed insignificant/unintelligible…”) or a feeling of being on the verge of an important breakthrough, increasing the sense of meaning. Some individuals report an enhancement of cognitive abilities, which they attribute to their newfound awareness. Approaching this “revelation”, which ultimately leads to the development of delusions, is marked by heightened emotionality, ranging from excitement over an impending discovery to anxiety and fear of something uncontrollable [5].

What makes aberrant salience experiences unique in psychosis is their persistence in the absence of sustaining stimuli. This characteristic has led to aberrant salience being considered a predisposing factor during the prodromal phase of psychosis and in ultra- high-risk (UHR) individuals. It is associated with various psychopathological disorders, particularly thought disorders [6].

Parnas and colleagues have explored the concept of Anomalous Self-Experiences (ASE), which serve as indicators of psychotic vulnerability and partially overlap with basic symptoms (BS) [7,8]. An anomalous self-experience can be defined as a pervasive or recurrent alteration in the perception of oneself as an agent capable of acting, experiencing, and existing in the world. These symptoms have been incorporated into a clinical staging model, in which the development of a psychotic episode progresses through increasing levels of symptom severity and intensity (ASE/BS → Attenuated Psychotic Syndrome (APS) → Brief Limited Intermittent Psychotic Symptoms (BLIPS)). In this model, the pathological attribution of salience to internal and/or external stimuli is present from the earliest stages [9].

This review will explore the characteristics of this prodromal period of psychotic vulnerability, which typically precedes the full-blown manifestation of psychopathology—referred to as the At-Risk Mental State (ARMS). It will further examine how this prodromal phase is psychopathologically related to aberrant salience and how this relationship can be leveraged as a screening tool for psychotic vulnerability. This perspective offers an additional valuable resource for Mental Health Centers, aiding in the early identification of individuals at risk of developing psychosis.

The core thesis of this work is the concept that AS represents a transdiagnostic, mechanistic marker of psychotic vulnerability and a viable target for early diagnostic and preventive interventions, particularly within UHR populations. This allows the mechanistic anchoring of psychotic onset in aberrant salience via dopamine dysregulation, glutamate-GABA imbalances, and altered salience network connectivity; conceptual bridging of clinical staging, basic symptoms, and psychotic-like experiences under the aberrant salience umbrella, clarifying their continuum nature; diagnostic justification the inclusion of salience-based psychometric tools as early detection instruments; arguments for early intervention (integrating neurocognitive and neuroimaging markers of aberrant salience can enhance transition risk prediction and support targeted therapeutic strategies); and addressing both state and trait vulnerability through a framework that links aberrant salience to cognitive deficits, neurodevelopmental disruptions, and interactions between genes and environment.

## 2. Analysis of Early Psychotic Symptoms Contributing Factors to Aberrant Salience

Among the established certainties of medical science is the role of genetics and the environment in which an individual lives. These two elements are not independent of one another but rather regulate and modify each other reciprocally. However, understanding how these two components interact is not easily achievable. Although research is still far from identifying the precise causes of psychosis, interest in this subject remains strong due to the potential role that understanding the mechanisms underlying the disorder’s genesis could have in its prevention.

The linear gene-phenotype model, which posits that a single gene or a group of genes is solely responsible for a given phenotype, has proven insufficient. Attempts to identify genes directly involved in the onset of psychotic spectrum disorders (the “main effects approach”) have been unsuccessful [10,11]. At the same time, epidemiological studies have observed a recurring presence of environmental risk factors among groups of patients with such disorders, including urbanization levels, immigration, substance abuse exposure (particularly cannabis), and trauma [12]. Consequently, attention has shifted towards how genetic and environmental factors interact. This has led to the development of the Gene × Environment (G × E) model, which does not establish a direct causal link for individual genes or environmental factors but rather emphasizes their synergistic co-participation in determining psychosis. According to this model, genes indirectly influence the disorder by regulating physiological pathways, thus determining the risk of developing a psychotic spectrum disorder rather than acting as its direct cause [13]. Genes can modify susceptibility to specific risk factors, explaining, for instance, why only a subset of individuals exposed to trauma or cannabis use develop a mental disorder [14,15]. Conversely, environmental factors can directly influence DNA sequences through de novo mutations or DNA methylation, leading to epimutations and altered gene expression [14,15].

The central role of genetic factors is not a novelty in psychiatry, where it is well established that psychotic disorders cluster within families and that having a first-degree relative with the condition represents the most significant risk factor for developing the disorder [16]. Adoption and twin studies have reinforced the idea that familial recurrence is due to genetic susceptibility [16]. Among individuals with schizophrenia who were adopted at birth, a higher incidence of the disorder has been found in their biological families rather than in their adoptive families [17]. Moreover, twin studies indicate that monozygotic twins, who share the same genetic material, are more susceptible to developing the disorder than dizygotic twins, who share only half of their genetic material. The estimated heritability based on such studies is approximately 92% [18]. These high heritability values have fueled expectations for linkage and association studies, though these have yet to yield solid and reproducible results [19].

Nevertheless, far from abandoning the issue, newer genome-wide association studies (GWAS) have identified both “common” and “rare” genetic variants associated with psychotic disorders. Single nucleotide polymorphisms (SNPs), or “common” variants present in at least 1% of the general population, are individually linked to a low risk of developing the disease but exert a substantial impact in a synergistic manner [20]. The results suggest that the genetic influence on such disorders arises from the cumulative effects of hundreds of thousands of small genetic variations across the genome [21]. Some identified variants fall within predictable genes, such as DRD2, which encodes the dopamine D2 receptor, the primary target of antipsychotics [22,23]. However, less predictable associations have also emerged, such as a strong linkage between psychosis and the major histocompatibility complex (MHC) locus on chromosome 6, an area of the genome known for its role in immunity. This association is partially explained by multiple alleles of the complement component C4 genes, which produce varying levels of C4A/C4B expression in the brain. Higher C4A expression has been linked to an increased risk of schizophrenia, as well as antipsychotic treatment response [24,25,26,27]. Notably, C4 expression in neurons has been shown to mediate synaptic pruning during neurodevelopment in animal models, highlighting a critical role for the immune system and complement pathways in the pathogenesis of psychotic disorders [28,29].

Alongside SNPs, rare copy number variations (CNVs) and deletions are more prevalent in affected individuals compared to controls [30]. CNVs, which are microdeletions or microduplications ranging from 1 kb to 2 Mb in size, represent a rare form of polymorphism in the genetics of these disorders, each occurring in less than 1% of patients [31]. However, CNVs are individually associated with a greater disease risk than SNPs [31,32,33]. For instance, a specific deletion on chromosome 22, which causes 22q11.2 deletion syndrome, is linked to an elevated risk of psychosis, with one in four affected individuals developing schizophrenia [34]. Recent studies showed recurrence of NRXN1 and ABCB11 disruptions in patients diagnosed with Schizophrenia, suggesting their role as a genetic risk factors [35]. However, this deletion is not specific to schizophrenia but is also associated with other medical and psychiatric conditions [36,37]. Schizophrenic patients with CNVs tend to exhibit more severe cognitive deficits and additional neurodevelopmental symptoms, such as facial dysmorphisms and short stature [37]. Genome sequencing studies have recently identified variants that may alter protein function and be associated with schizophrenia [30,31]. Psychosis-associated CNVs appear to be more prevalent in genes involved in neurodevelopment, such as neuregulin [38], and have been identified in individuals from families with a strong genetic predisposition [39]. Thus, GWAS findings suggest that rare protein-altering variants may contribute more significantly to psychosis risk than common variants, though overall risk likely results from their combined effects.

It is also noteworthy that polygenic analyses of multiple disorders suggest that genetic loci associated with psychosis are also implicated in other mental or physical conditions. The greatest overlap is observed between schizophrenia and bipolar disorder, major depressive disorder, and autism [40,41,42,43]. Interestingly, there is also an increased risk of inflammatory bowel disease among individuals carrying schizophrenia-associated genetic variants, further supporting the role of the immune system in the disorder’s etiology [44,45]. In summary, the genetic component of psychosis is highly polygenic, results from a combination of rare and common variations, and overlaps with other disease categories.

The relative contribution of environmental risk factors to the onset of psychosis remains a subject of intense debate in psychiatric research. Recent studies suggest a lesser role for genetics and a greater influence of environmental factors, with epidemiological research confirming strong associations between exposure to multiple environmental stressors and psychosis [46,47]. Many environmental risk factors increase susceptibility depending on the developmental stage at which they occur [12]. Others, such as parental socioeconomic status, sociodemographic factors (e.g., educational attainment and race/ethnicity), and income disparity, tend to remain stable across development and have broad implications for lifelong health [48,49,50,51].

Among the most significant risk factors identified there are prenatal and perinatal complications, childhood trauma, substance abuse (especially cannabis), and urbanization [52,53,54,55]. These environmental factors interact with genetic vulnerabilities to shape individual risk according to the Gene × Environment (G × E) model. Precisely because environmental factors play a fundamental role in the development of a subsequent psychosis framework, it is crucial to address them through policy interventions aimed at reducing the presence of these risk factors at the societal level. This includes efforts to improve socio-economic conditions and reduce disparities and inequalities, as well as policies that ensure the dignified treatment of the human person.

Moreover, public information campaigns about the potential risks associated with the use of substances such as cannabis are becoming an essential component of prevention strategies. Adolescence is a critical period of neurodevelopment, characterized by marked synaptic pruning and increased myelination [56]. Furthermore, the endocannabinoid system appears to be involved in regulating key neurodevelopmental processes, suggesting that the introduction of exogenous cannabinoids during adolescence could interfere with normal brain maturation, in addition to significantly increasing the risk of developing psychosis.

Aberrant salience—defined as the inappropriate attribution of significance to otherwise neutral stimuli—has been proposed as a key factor in the prodromal phase of psychosis and in ultra-high-risk (UHR) individuals, particularly influencing thought disorders [6].

## 3. Bonn’s Group Contribution: The Basic Symptoms

Over the last two decades, the traditional model of psychotic illness has undergone a major process of revision that has led to the abandonment of the static dichotomous view of psychotic disorder in favor of a conceptualization that sees psychosis as an extended phenotype along a continuum between clinical and non-clinical manifestations, where at one end lies schizophrenia considered as the most disabling and complete form, in the middle non-psychotic mental disorders that may have in the course of their clinical history psychotic-like manifestations or frank symptomatology, and at the other extreme psychotic-like experiences, experienced by the general healthy population, but not requiring long-term care [57].

As noted by Van Os et al. [58], although schizophrenia represents the most disabling and studied form of the much broader and more varied spectrum, the prevalence of psychotic-like experiences (PLE, a risk marker for psychosis or susceptibility to other psychiatric pathologies) in the general population would be much higher [59]: indeed, thinking about states of derealization, certain delusional beliefs or pseudo-hallucinations, or temporary hallucinations experienced in some forms of obsessive-compulsive disorder, it is clear how these can also be experienced by individuals not affected by typically psychotic disorders [60]. Such psychotic-like experiences are also be transiently experienced by the general Western population, at a rate of 25% in the American population [61] and 17.5 per cent in the Netherlands [58], or hypnagogic and hypnopompic hallucinations in 40 per cent of the sample of an Italian-English cohort study [62]. Recent publications highlight how PLEs could be useful in identifying the risk of future psychiatric, psychotic, and non-psychotic disorders at an early stage [63]. Their presence in fact increases the risk of evolution towards any psychiatric pathology by a factor of three, while it increases the risk for psychosis by a factor of four [64]. However, PLEs could also be considered non-specific markers of stress [65]. As these are both indicative of mental distress and life adversity, it is useful to study psychotic-like experiences in adolescents [66].

Within this new dimensional perspective, there are certain subclinical disorders of a subjective nature called basic symptoms, concerning the affective, cognitive, verbal, somatic-perceptual, motor, and vegetative dimensions. They can be found both in individuals at risk of developing a schizophrenic spectrum disorder and in the intervallic and post-acute phases of the disorder, which have been much studied in the field of research and clinically [67]. This model, developed by the German Bonn group led by Gerd Huber, offers an interpretation of the experience of psychotic vulnerability, in which the patient has subjective experiences, that can be assessed using the Bonn Schedule for the Assessment of basic symptoms, or the BSABS [68]. These experiences, although strange and confusing, are not yet of the same magnitude as the classic productive symptoms (hallucinations and delusions), and are disorganized and negative [69].

The peculiarity of such manifestations is that they tend to anticipate the onset of the full-blown manifestation and then gradually fade away. Moreover, these are distinguished from negative symptoms in their current understanding because they represent dysfunctional mental and behavioral responses that can be observed by others, and from positive symptoms that stripped of insight (still present in BSs) are experienced as real [70,71]. The psychopathological transition state which the subject undergoes consists of three phases specifically [68,72,73]. Initially, the subject experiences a ‘basal irritation’, in which there is an increase in emotional tension and cognitive complexity of the experience as the basic symptoms intensify and become more pervasive. This experience interferes with daily life, leading the person to perceive their surroundings, their thoughts, their body and their actions as less natural and fluid, materializing in an experience of derealization, depersonalization, and perplexity.

This is followed by ‘psychotic externalization’, in which the anomalous and disturbing sensations experienced by the person as foreign are attributed to the action of unknown external agents. This process represents the first attempt to justify what the subject cannot correctly interpret, in order to solve the confusion surrounding the experiential transformation. The final phase of ‘content concretization’ takes place when there is certainty about how, who, and why such a manipulation is taking place. Now the delirious design acquires a complete and well-defined descriptive value, with a high degree of explanatory coherence, cognitive complexity, and emotional tension, leading to elaboration upon the delusional thought, which may be, for example, of a persecutory type: thus, a new coherent order of the previous experiences of the shattering of the self is realized [74]. It is in this sequence of events that the psychopathological dimensional continuum of psychosis is realized, and where early and punctual intervention is a must, especially in those in whom such distress progressively resurfaces, leading them to seek help from a mental health service (help-seekers) [75].

Basic symptoms (BSs), being subjective, remain predominantly private and belong only to the person concerned, so that they are hardly observable by others, although they may manifest in coping strategies adopted by the patient, such as avoidance behavior and social withdrawal in response to the symptoms themselves [76]. These are different from overt positive psychotic symptoms, since, stripped of insight, they are experienced by the patient as real, normal, and incontrovertible thoughts and feelings [70]. On the contrary, BSs are immediately and spontaneously recognized by the person as extraneous and as alterations of his or her mental processes, associated with this perception of internal change: the person is in fact aware of the incoherence of these thoughts, so much so that some experiences may even be inexplicable to him or her [77]. Faced with this experience, the person may be able to express what he or she is experiencing autonomously, but very often needs help from a practitioner through guided questions in order to be able to provide a more in-depth description [78]. Recognition of BSs may sometimes be difficult due to their gradual fading as the psychotic illness progresses or because they are masked by the psychotic symptoms themselves during the acute phase. Klosterkötter et al., in their 2001 Cologne Early Recognition (CER) study, found that these symptoms were important in predicting an evolution of the clinical picture into full-blown psychosis [79].

Basic symptoms include perceptual impairments (Cognitive Perceptive, COPER) and cognitive disturbances (COGDIS) [77], shown in Table 1.

According to the model offered by Huber and Gross in 1989, the first symptoms would manifest themselves through three developmental levels: on the first level, there are the basic “uncharacteristic symptoms” that affect pulsion, abolition and affection, concentration, and memory; on the second level we have the “characteristic symptoms”, which are qualitatively peculiar, especially from a cognitive, perceptual, and motor point of view; finally, on the third level, there are the “productive psychotic symptoms” [76]. At the beginning of the first level, BSs would gradually increase in number and intensity, evolving into frank psychotic symptoms for the most part. Sometimes, first- and second-level BSs would go into complete and spontaneous remission before reaching the full-blown phase. These symptomatic phases, which have not progressed into a frank psychotic episode, represent the true prodromal states and are called outpost syndromes, as they precede the next prodrome in an already pathologically vulnerable person. The transition from the second to the third level is facilitated by daily stressors, which result in an overload of information for the person to process, or the overload of coping strategies and personal resources [72,77].

## 4. Aberrant Salience, Bayesian Theory and Reality Testing Inference

The study of the nervous system is framed as a “system of relationships”, where neurons exist due to the exchange of information between them, allowing the nervous system to perform complex functions [80]. Psychiatric pathologies are seen as network pathologies, and dopamine plays a fundamental role as a “mediator of pleasure and reward”, influencing decision-making and response anticipation, independent of valence. At the cortical level, pleasure can be “consumed” only when it is meaningfully attached. This leads to the “motivational salience hypothesis” [81], proposing that dopamine mediates the attribution of salience in the brain. The disruption of the mesolimbic and mesocortical dopaminergic system, particularly involving the prefrontal cortex (PFC) and nucleus accumbens (NAc), is central to the physiopathology of psychosis, labeled as i-RISA (impaired response inhibition and salience attribution), where faulty salience attribution leads to inappropriate behaviors [82].

**Figure 1:**
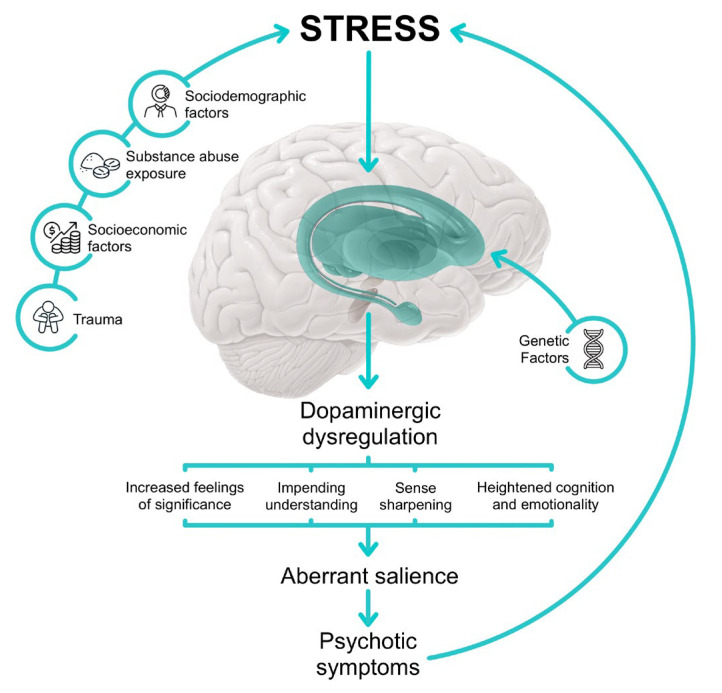
Illustrated pathway leading to aberrant salience.

Psychosis is characterized by a disturbance in reality testing, often marked by delusions (false beliefs resistant to logic) and hallucinations (hyper-reflexivity), manifesting as a result of dopaminergic dysregulation. In psychosis, dopamine misguides salience attribution, causing individuals to give inappropriate significance to both external stimuli and internal representations. The continuous misattribution of salience, even in the absence of concrete external stimuli, is a hallmark of psychotic experiences, as represented in Figure 1. Delusions represent cognitive explanations constructed by the individual to restore coherence to their perception of the world, while hallucinations are the result of misattribution of salience to perceptions or memories. Delusional thinking provides the individual with a sense of relief from earlier anguish, as they form a new cognitive and behavioral framework that validates their beliefs. These patients may exhibit altered probabilistic reasoning, perceptual distortions, and magical thinking, with psychotic phenomena originating from neurobiological and psychodynamic abnormalities [83].

Bayesian theory focuses on how the brain “explains and interprets” the world, proposing that the brain makes inferences about the world based on internal schemas, integrating sensory inputs with prior predictions. Perception, in this context, is seen as a result of the integration of “a priori” predictions and “a posteriori” sensory input. Aberrant perceptual inferences in conditions like schizophrenia may arise from biological anomalies in the neuronal representation of these internal schemas or inputs [84]. According to the theory, prediction errors occur when there is a discrepancy between predictions and actual sensory experiences. The brain processes these errors through various regions, adjusting predictions at lower hierarchical levels based on these errors. Hallucinations may occur when the brain overly emphasizes predictions, ignoring sensory data that contradicts them. This theory provides an explanation for the brain’s faulty inference processes, resulting in psychotic symptoms.

### From the Definition of Prodrome to At-Risk Mental States and Help Seeking

In recent decades, there has been a paradigm shift in mental health research and clinical practice, focusing more on identifying and managing early signs of distress that may signal the onset of mental disorders. This shift emerged from research and observational studies, highlighting the potential of early intervention to prevent psychotic disorders. A critical period during late adolescence and early adulthood, when prodromal states are still malleable, has been identified as a key focus area [85]. This period, often documented through retrospective assessments in mental health services, is crucial for the development of early intervention models aimed at addressing first psychotic episodes and evaluating individuals at high risk of psychosis.

The necessity for early intervention became clear due to the delay between the onset of psychotic symptoms and effective treatment, which has been shown to result in worse long-term outcomes [86]. Psychiatric disorders often have preceding clinical conditions marked by non-specific symptoms, such as anxiety, depression, sleep disturbances, and subtle psychotic symptoms, referred to as prodromal states. Sullivan (1994) proposed that the onset of schizophrenia could be averted through early intervention during this prodromal phase, a hypothesis supported by studies like the “ABC Study”, which showed that 70% of psychoses were preceded by a prodromal phase [87].

The concept of risk in this context is probabilistic, relating to the likelihood of an event causing harm, such as the onset of a psychotic state. Cross-sectional studies have identified “static” risk factors that are difficult to modify, including genetic factors, early traumatic events, and socio-demographic factors (age, gender, urbanization), while longitudinal studies have highlighted “dynamic” factors that can be intervened upon, such as stigma, substance use, and challenges related to acculturation after immigration.

Since the 1990s, psychiatric research has increasingly focused on identifying models to predict the onset and development of psychotic disorders. Conditions with a high risk of transition are known as “At-Risk Mental States (ARMS)”, “Clinical High Risk (CHR)”, or “Psychosis Risk Syndrome (PRS)” [88,89]. The Australian group at the PACE Clinic developed the ultra-high-risk (UHR) criteria for help-seeking individuals aged 15–25, identifying three high-risk states: Genetic Risk and Deterioration (GRD) syndrome, characterized by a family history of psychotic disorders and recent psychosocial deterioration; the Attenuated Psychotic Symptoms (APS) group, marked by less intense or frequent psychotic symptoms; and the Brief Limited Intermittent Psychotic Symptoms (BLIPS) group, with fully formed but brief or intermittent psychotic symptoms.

A meta-analysis published in 2021 reported a mean prevalence of 1.7% (95% confidence interval (C.I.) = 1.0–2.9%), which is in line with the point prevalence of psychotic disorders (0.389%) [90]. However, the authors themselves highlighted that the results of the studies they referenced were rather conflicting [91]. This challenge in assessing prevalence in the general population is attributable to various factors, including the fact that UHR criteria were developed exclusively for assessment in help-seeking individuals and clinical populations [91]. In clinical samples, the prevalence was found to be approximately 19.2% (95% C.I. = 12.9–27.7%), more than ten times higher than that found in the general population.

This confirms the specificity of the UHR criteria for the clinical population. The results align with current empirical knowledge: the prevalence of psychosis corresponds to approximately 22.9% (0.389/1.7) of estimated CHR-P cases in the general population, which roughly matches the transition estimates in CHR-P patients (the psychosis risk is about 20% after two years). The prevalence in the general population is low, while in the clinical population, it is relatively high. This, according to de Pablo and colleagues [91], may indicate that the risk condition often goes unnoticed by non-specialized clinicians.

Regarding transition rates, the most recent meta-analysis [92] reports the following cumulative transition risks to psychosis: the quantitative risk level increases from 9% at 6 months to 25% at 3 years, reaching 26% by the fourth year of follow-up, thus demonstrating that the peak risk occurs between the third and fourth year. Additionally, the results revealed that the risk continues to slowly rise, reaching 35% over 10 years. Consequently, transition rates are observed to be elevated during the initial months following CHR assessment, with 80% of transitions occurring within the first two years.

In their respective studies, Fusar-Poli [93,94] and de Pablo [92] draw attention to several significant aspects. They observe that the average age of the samples is approximately 20 years, with a range from 12 to 49 years, thus indicating that the paradigm predominantly focuses on the young adult population. Despite this, age does not appear to significantly moderate the risk, which remains relatively consistent among children, adolescents, and adults. Another key observation is the higher prevalence of males in these studies, accounting for approximately 58% of the sample, which aligns with the higher incidence of psychotic disorders observed in men. Individuals presenting with brief limited intermittent psychotic symptoms (BLIPS) appear to be at a higher risk of transitioning to a more severe condition, particularly if their symptoms are recurrent or involve severely disorganizing features. The clinical high risk for psychosis (CHR-P) population also shows a high level of comorbid disorders, with the most commonly reported conditions including depressive disorders (41%), anxiety disorders (15%), and substance use disorders (25%). Furthermore, 44% of individuals report having one or more diagnoses of personality disorders, and 66% of cases report suicidal ideation, with 49% engaging in self-harm and 18% attempting suicide.

In relation to the transition to psychosis, 73% of individuals in the CHR-P group ultimately develop schizophrenia, which is considerably higher than the 11% who develop affective psychosis and the 16% who transition to other forms of psychosis. It is noteworthy that negative symptoms, despite their presence and their documented negative impact on patient functioning and prognosis [95], are frequently not given adequate consideration in these studies. Prognostic considerations also emphasize the differences in risk among the three CHR-P subgroups. After 48 months, the cumulative transition risk is highest for the BLIPS group at 38%, followed by 24% for the APS (attenuated psychosis syndrome) group, and 8% for the GRD (genetic risk and deterioration) group, which does not exceed the transition rates observed in non-CHR-P help-seeking control groups. However, other studies have found no significant long-term differences between the three subgroups in terms of severity, functioning, or outcomes beyond disorders within the schizophrenic spectrum [96]. Another association found is between greater severity and duration of symptoms (both APS and BLIPS) and an increased risk of transition, highlighting the need for timely intervention. Consequently, the “wait and see” approach is not recommended on clinical grounds, particularly in view of the fact that unfavorable trajectories (development of psychosis, relapses, absence of remission) account for 57.1% [91,97,98].

In clinical practice, the identification and subsequent treatment of ARMS patients also relies on a model taken from general medicine, the Clinical Stage Model. This model considers psychotic disorder to be a continuum, with each intervention aimed at promoting recovery while simultaneously preventing progression to the next stage of the disorder. This approach offers effective and less invasive interventions at earlier stages compared to those required for more advanced stages of the disorder. The stages are defined by the severity of symptoms, distress, and resulting disability, as listed in Table 2 [99].

Following the implementation of this model, one of the primary concerns was that a significant proportion of individuals identified with the UHR criteria did not develop psychosis within 12 months. This observation led to considerations regarding the potential for stigmatization associated with a psychosis diagnosis. Additionally, the transition rate appears to be lower than initially hypothesized, with a decline in transition rates observed in services that have adopted early intervention principles [88,89]. This has led to questions regarding the positive predictive value of the UHR criteria, as the likelihood of transitioning to a psychotic disorder within a short to medium term is less than 50% for UHR patients treated, potentially raising concerns about overtreatment. To reduce the number of false positives and improve the positive predictive value (PPV), a study was conducted on a sample of 104 individuals at risk of psychosis. In addition to the UHR criteria, the study examined other clinical or demographic factors that could be used in this regard [100], identifying four clinical predictors of transition: attenuated psychotic symptoms combined with genetic risk, long duration of symptoms, poor social functioning, and attention deficits. The presence of at least one of these factors results in an 80.8% PPV, 60% sensitivity, and 92.6% specificity.

The European Prediction of Psychosis Study (EPOS) proposed a model with six variables, including positive symptoms, bizarre thinking, sleep disturbances, schizotypal personality disorder, scores on social functioning scales (GAF), and years of education [101]. Furthermore, the integration of baseline symptoms with the UHR criteria has been demonstrated to enhance PPV [102], with a higher conversion rate to psychosis at 48 months in patients who, in addition to meeting the UHR criteria, presented cognitive baseline symptoms (COGDIS). It has also been noted that the presence of psychotic experiences and sub-threshold psychotic symptoms is associated with poorer outcomes and an increased risk of suicide [103]. All individuals diagnosed with ARMS also present with anxiety or mood disorders that require intervention [104]. This observation gives rise to the hypothesis that early intervention may be beneficial in a number of ways. Firstly, it would allow for timely diagnosis and prevention of full-blown psychosis. Secondly, it would allow for treatment of other concurrent mental health disorders, for which individuals diagnosed with ARMS often meet the criteria, thus improving their overall outcome.

## 5. Neurobiological Hypotheses and Connectivity Alterations

In order to enhance comprehension of the etiology and manifestations of neuroanatomical alterations that occur during the early stages of psychosis development and later on, integration of evidence derived from pharmacological therapies, neuroimaging, and post-mortem investigations is necessary. This has led to the hypothesis that psychotic disorders are linked to four primary pathophysiological mechanisms: altered dopaminergic, glutamatergic, and serotonergic neurotransmission, associated with a pro-inflammatory state, which interact with each other and are likely causally related. The well-known effect of certain substances of abuse confirms the role of these neurotransmitters: psychostimulants such as cocaine and amphetamines, by releasing dopamine and stimulating the D2 dopaminergic receptor, primarily cause auditory hallucinations and paranoid delusions; dissociative anesthetics like phencyclidine (PCP) and ketamine are NMDA receptor antagonists, which are responsible for visual hallucinations and paranoid delusions, as well as dissociative states; and hallucinogens or psychedelics like LSD (lysergic acid diethylamide) and psilocybin, 5-HT2A agonists, induce visual hallucinations or mystical delusions [105]. These pathways interact with each other on multiple levels, thereby explaining the great heterogeneity with which psychotic symptoms manifest in various psychiatric and neurological diseases [106,107]. For example, auditory hallucinations and paranoid delusions in schizophrenia are not the same as those occurring in Parkinson’s disease and dementia-associated psychoses. The latter are primarily associated with visual hallucinations and delusions of jealousy or persecution, often with maintained insight, and are effectively treated with 5-HT2A antagonists, such as Pimavanserin, without D2 dopamine-antagonistic effects [108].

### 5.1. Dopaminergic Hypothesis

Following the identification of the antipsychotic properties of Chlorpromazine in the 1950s, it has been demonstrated that the blocking of dopaminergic D2 receptors can result in a reduction in positive symptoms in patients diagnosed with schizophrenia. Specifically, the well-known and extensively studied pathophysiological mechanism is associated with the hyperactivity of the mesolimbic dopaminergic pathway, which originates from the ventral tegmental area and projects to limbic regions, particularly the nucleus accumbens. This pathway has been hypothesized to be responsible for the manifestation of classic psychotic symptoms, including delusions, frequently of a paranoid nature, and auditory hallucinations. Conversely, negative symptoms and cognitive deterioration have been attributed by numerous scholars to dopaminergic deficits in specific frontal regions, notably the dorsolateral prefrontal cortex (DLPFC), which are projections of the mesocortical dopaminergic pathway, originating from the same tegmental area as the aforementioned pathway.

This deficit could be primary or secondary to issues with the mesocortical pathway, where the inhibition of dopamine release could be due to serotoninergic hyperactivity [109]. While initial attention focused on postsynaptic DA receptors, more recent PET studies using (18)F-DOPA as a tracer have shown that the major dysfunction occurs presynaptically, with increased dopamine synthesis and release. This increase in (18)F-DOPA uptake has been observed during the ARMS period and has been shown to be a predictor of the development of clinically manifest psychosis [110,111,112]. The complexity of the situation is further compounded by the existence of five different types of dopaminergic receptors: D2 located at the striatal level, D3 and D4 at the limbic level, and D1 in the cortex and basal ganglia. This heterogeneous distribution, in addition to highlighting the complexity underlying these manifestations, also explains the problems related to pharmacological therapy, particularly with first-generation antipsychotic molecules: their antipsychotic effect, as mentioned earlier, is mediated by blocking the mesolimbic pathway but at the cost of blocking the mesocortical pathway, thus enhancing (and worsening) negative symptoms. Furthermore, these medications have been observed to block two additional pathways: the nigrostriatal, resulting in classic extrapyramidal effects and, via up-regulation, the occurrence of tardive dyskinesia, frequently irreversible, and the tuberoinfundibular pathway, leading to galactorrhea.

### 5.2. Glutamatergic Hypothesis

A more recent focus has been on another neurotransmitter, glutamate, which appears to play a primary role in the pathophysiology of psychotic disorders [113]. It has been hypothesized that dysfunction of the receptor to which it binds, the N-Methyl-D-Aspartate (NMDA) receptor, located in primary and secondary cortical glutamatergic neurons, leads to a consequent dysfunction of GABAergic interneurons [114]. The loss of this inhibitory tone, which is normally regulated by GABA, on secondary glutamatergic neurons results in their increased activation asynchronously, with excessive firing of dopaminergic neurons in the mesolimbic pathway [115]. This assertion is corroborated by studies on NMDA receptor antagonists, ketamine and PCP, and by cases of autoimmune encephalitis, where the production of antibodies against NMDA receptors can generate a condition indistinguishable from schizophrenia [116]. During neural development, this receptor plays a central role in synaptic plasticity, laying the foundation for the development of higher cognitive functions such as memory and learning [117].

During neurodevelopment, the NMDA receptor, which possesses a heterotetrameric structure comprising a fixed NR1 subunit and two variable NR2 subunits, undergoes continuous changes that alter its structure, leading to maturation through the replacement of some subunits with others that are structurally different [118,119,120]. With maturation of the receptor, its functioning and physiological properties also change, rendering it more sensitive to environmental stimuli and able to initiate appropriate neuronal electrical activity at the right moment [118]. It is important to note that this receptor switch does not occur uniformly across all brain areas. These differences in the timing of receptor maturation could coincide with vulnerable periods for psychotic disorders, when the individual is particularly at risk from external factors such as hypoxia, perinatal stress, infections, inflammations, substance abuse, or social isolation [118,119,120].

For instance, during pregnancy, there is an increase in NMDA receptor levels, rendering the fetus more susceptible to external insults [121]. The hypothesis that risk factors delay or interrupt the receptor switch in specific brain areas has been advanced, with the result that proper receptor maturation is prevented, thus impairing long-term depression and potentiation [122]. In the first case, weak and rarely used connections are eliminated, while in the second, those that are more frequently used are enhanced. This process, which reaches its physiological peak during adolescence, is known as synaptic pruning. Alterations to this process have the potential to contribute to the onset of schizophrenia and the “disconnection” characteristic of the disorder [123]. The progressive changes in white and grey matter during adolescence, which are associated with changes in brain functioning, can have a significant impact on the brain dysfunction observed in the prodromal phases of psychosis. This can result in a progressive accentuation of cognitive decline, which eventually leads to the development of overt psychotic symptoms [124]. The compromise of the glutamatergic pathway, due to decreased NMDA receptor functioning, leads to hypofunction of inhibitory GABAergic interneurons, hindering the synchronization of neuronal firing at the level of pyramidal neurons. A decline in neuronal synchrony has been demonstrated to result in cognitive impairment. It is noteworthy that this compromise is corroborated by post-mortem studies demonstrating a reduction in a subset of GABAergic interneurons, namely chandelier cells containing parvalbumin, in patients diagnosed with schizophrenia [125]. Furthermore, a decline in enzymes involved in GABAergic transmission, such as GAD67 (glutamic acid decarboxylase) and GAT1 (GABA transporter), has been observed. A further post-mortem study revealed a shift in expression from GAD25 to GAD67 and from NKCC1 to KCC2 in the prefrontal cortex and hippocampus of patients with schizophrenia. The former increases GABA synthesis, while the latter shifts neurotransmission from excitatory to inhibitory [126]. This assertion is further substantiated by mouse models demonstrating that the same glutamatergic afferents from the hippocampus to the nucleus accumbens exert a robust excitatory effect on striatal dopaminergic neurons [127]. Consequently, diminished activation of the NMDA receptor results in augmented DA release within the nucleus accumbens, precipitating the emergence of positive symptoms such as delusions and hallucinations. Consequently, the dysregulation of glutamate, concomitant with the loss of GABAergic signaling, may represent pivotal mechanisms in the etiology of psychosis.

## 6. Aberrant Salience and Psychotic Ideation Research Methodologies

### 6.1. Neuroimaging and Aberrant Salience

Since the mid-20th century, there has been a concerted research effort to identify the morphofunctional alterations at the brain level that underlie the corresponding clinical changes. This deterioration, although manifesting only when the pathology is clinically evident, is thought to begin subtly several years before. The earliest research in this area can be traced back to the 1950s, when pneumoencephalography revealed a moderate enlargement of the ventricular system in a group of schizophrenic patients with cognitive deficits [76]. More recently, neuroimaging results from a large meta-analysis conducted on 18,000 individuals showed a slight but significant decrease in intracranial volume in both chronic and first-episode treatment-naive psychotic patients [128]. The maximum size of the brain is reached around the age of 13, after which growth ceases. This is due to developmental anomalies, which result in significant losses of both grey and white matter [129]. However, while the former seems to be more compromised in chronic patients than in treatment-naïve patients, the latter loss appears equally represented in both groups [130].

Indeed, the loss of white matter has been shown to be quantitatively similar in first-episode psychosis and chronic forms of the disease, with no progression in the latter. This finding aligns with the results of twin studies, which suggest that this reduction in white matter is more likely to be a genetic risk factor for developing the disease than an effect of the disease itself [131]. In contrast, grey matter undergoes a progressive deterioration as the disease progresses, manifesting predominantly as a reduction in cortical thickness that is associated with outcome [132], adherence to therapy [133], cannabis use [134], and psychotic relapses [135]. This evidence confirms that, although some alterations in brain development worsen after the first psychotic onset, other changes occur during the preceding years [136]. All this seems to be the direct consequence of altered connectivity between different brain areas [137,138,139].

Diffusion Tensor Imaging (DTI) is a technique used to study this, utilizing fractional anisotropy, a unique directionality dependent on the diffusion of water molecules, characteristic of homogeneous anatomical structures such as white matter tracts, to create 3D images. This technique has highlighted differences in the uncinate fasciculus, arcuate fasciculus, superior longitudinal, and the anterior part of the corpus callosum, suggestive of axonal or glial damage and/or increased free water concentration [140,141,142,143,144,145]. A reduction in anisotropy has been correlated with a higher degree of cognitive dysfunction and a poor response to potential antipsychotic treatment [146,147,148]. While white matter alterations occur at the level of associative fibers, gray matter damage does not show a uniform distribution and is highly variable [132]. The most affected areas in grey matter in patients evaluated after a first psychotic episode are the frontal and temporal regions, including the insula, parahippocampal area superior temporal gyrus, and anterior cingulate gyrus [149,150,151,152,153]. The majority of longitudinal studies have demonstrated that following the initial episode, neuronal loss persists, accompanied by cortical thinning, predominantly in the frontal and temporal regions. This has been associated with deteriorating clinical outcomes, particularly in terms of cognitive ability [149,151,154].

A study by Mallikarjun et al. (2018) [155] examined functional connectivity in patients experiencing their first episode of psychosis with frequent auditory verbal hallucinations, using brain regions identified during real-time symptom capture. The findings suggest increased connectivity between the left insula and both the cerebellum and angular gyrus, as well as between the left claustrum and both the cerebellum and postcentral gyrus. Additionally, there was reduced connectivity between the left claustrum and the left insula in patients with auditory verbal hallucinations compared to healthy individuals. However, no significant differences in connectivity were found in auditory processing regions such as the superior temporal gyrus, memory-related areas including the parahippocampal and lingual gyrus, or the posterior regions of the default mode network, such as the precuneus and posterior cingulate cortex. These results suggest that auditory verbal hallucinations may arise from disruptions in communication between the salience network and the default mode network. The insula and angular gyrus appear to play a crucial role in this process, while dysfunction in the claustrum-insula complex may contribute to difficulties in evaluating the significance of sensory information. Additionally, altered cerebellar connectivity supports its role in prediction errors related to sensory perception, which may contribute to the experience of hallucinations [155].

A 2021 meta-analysis by Kowalski and colleagues [156] examined the neural correlates of aberrant salience and source monitoring in schizophrenia and individuals at clinical risk for psychosis. The study analyzed data from 1363 identified articles, excluding reviews, non-experimental studies, non-English articles, and those without participants with schizophrenia or at risk of psychosis, ultimately including 18 articles. Of these, seven fMRI studies of abnormal salience were identified, with an additional study found through a reverse search, giving a total of eight studies. Overall, the findings suggest that aberrant salience and source monitoring share neural mechanisms, particularly in the ventral striatum, insula, medial prefrontal cortex, and temporal gyri, with possible functional overlap in the anterior cingulate cortex, paracingulate sulcus, and hippocampus. This overlap may contribute to positive symptoms.

Dynamic functional connectivity studies, the aim of which is to augment the information obtained from functional brain scans by considering changes in network structures over time [157] with the potential of studying the complex functional organization of the brain, has shown that subjects with clinical high-risk (CHR) for psychosis present specific changes in the connections between the superior frontal gyrus and calcarine cortex and alterations in common with schizophrenic subjects in the supplementary motor area, para-hippocampal gyrus and postcentral cortex, thus finding patterns with similarities to healthy subjects than to patients diagnosed with schizophrenia [158].

Another model being studied to explain the complexity of change in brain connections is multilayer networks, in which changes in the various networks over time are represented. This model could be capable of integrating data that are constantly evolving in time and space, allowing the brain to be analyzed in a less static manner [159], in the dynamism that characterizes it, capturing instabilities, fluctuations, and patterns that can also characterize pathologies such as schizophrenia [160] and depression [161]. In a study from 2022 [162], this model was used to associate psychosis-like traits in healthy individuals with instability of connections in the Default Mode Network (DMN), a brain network related to internal thought and self-perception.

### 6.2. Psychotic Risk, Cognitive Impairment and Neuropsychological Assessment

One of the central aspects and causes of disability in psychotic spectrum disorders is neurocognitive impairment, which is observed at all stages of the illness and is the most important predictor of occupational and functional impairment in these patients. A reduction in cognitive performance is already observed in the prodromal phases of the illness and at the onset of psychosis, which typically occurs in adolescence and early adulthood, and remains a characteristic and stable feature of the illness.

To better understand how cognition is related to functional outcomes in psychosis, it seems essential to examine neurodevelopment. Based on the neurodevelopmental model of psychotic disorders proposed by Niendam and co-workers (2009) [163], the role of early and developmental interactions between the genome and the primary environment is evident, as mentioned in other sections of this paper. The onset of psychotic disorders must in fact be seen as a multifaceted and complex long-term process, starting from basic risk elements constituted by genetic factors to prenatal and perinatal pathogenic factors (hypoxia, infections, malnutrition, immunopharmacological incompatibilities) and early relational experiences. In fact, they can influence the developmental stages of the central nervous system, in particular the formation and migration of neurons during pregnancy, and then synaptogenesis, pruning, and apoptosis during brain maturation. Any alteration in these processes inevitably leads to morpho-functional abnormalities in the CNS, which in turn would underlie the alteration of cognitive-emotional processes, together with neuropsychological deficits in attention, memory, executive functions, and emotional regulation [164]. The resulting brain abnormalities may thus contribute to the manifestation of clinical symptoms, cognitive and psychosocial deficits associated with progression to full-blown illness in at-risk individuals. As previously observed in describing the environmental factors that may contribute to the development of schizophrenia, hypoxic complications during birth predict increased grey matter loss in these patients [165]. In fact, all the early changes that occur in the earliest stages of development, such as reduced synaptic plasticity and disruption of white matter integrity, as early brain vulnerabilities, can alter the synaptic pruning process that peaks during adolescence and, when altered, produces the anatomo-functional disconnection that is characteristic of this disorder [128]. These changes in white and grey matter translate functionally into progressive cerebral deterioration, which is responsible for the reduced functionality during the prodromal phase of psychosis. In fact, cognitive decline occurs not only during the active and overt phase of the disease, but also during the prodromal period in association with changes in brain function, making cognitive deficits a stable risk marker in high-risk populations [163].

Although the classical description of schizophrenic symptomatology refers mainly to positive or negative symptoms, the neurocognitive deficits observed are a central aspect of the disorder [166,167,168]. In fact, as noted above, these deficits are early and stable (typically even in cases of pharmacological resistance or remission), and they are observed to varying degrees across the spectrum of psychotic disorders, in people familiar with such disorders as well as in people with overt disorders and apparently good functioning. In the overt and more severe form of the disease, typically schizophrenia, there are multiple deficits in the various neuropsychological domains [169]: in particular, severe impairments have been observed in general cognitive functioning, especially in performance intelligence quotient (IQ) or, for example, in processing speed, which has been found to be significantly impacted independent of task complexity. Confirming the difficulties related to prefrontal functioning, impairments in executive functions were found in tasks of selective attention, planning, verbal fluency, and verbal and visuospatial working memory, or both verbal and non-verbal immediate and delayed declarative memory, typically in the acquisition phase [167].

Of lesser importance, but still relevant, are the deficits attributable to attention, non-declarative learning and basic language skills: what seems to be most impaired is the pragmatic and non-literal use of language [170,171,172], as well as associative learning [173]. Such difficulties also occur in emotion processing and social skills, with a strong association observed between difficulties in the area of theory of mind and functioning, highlighting a deterioration in what is termed ‘social cognition’ [174].

While what has just been described refers to the overt form of the disorder, one meta-analysis [175] has shown that a general mild to moderate impairment in neuropsychological functioning and social cognition is also evident in those considered at risk of developing the psychotic disorder, who show deficits compared to controls in most of the domains examined, including general intelligence, executive and frontal functions (such as control functions and working memory), visual and verbal memory, verbal fluency, attention, and social cognition. As in overt psychosis, an important cognitive domain that is impaired in UHR subjects is social cognition, i.e., the ability to interpret the behavior of others and formulate an adaptive response [176], resulting in increasing functional impairment during the sensitive developmental periods that occur during adolescence. One hypothesis is that this impairment is due to a deficit in emotion recognition in faces, which is present in psychosis as well as in individuals at high clinical risk [164,177]. The importance attached to deficits in social cognition and poor social functioning is such that they are considered trait markers that increase vulnerability to various risk factors [178].

The ability of an individual to act autonomously and make decisions by influencing their surroundings, which could be termed agency, has also been related to psychosis. In a 2019 study [179], carried out by Di Plinio and collaborators, an attempt was made to find out whether intentional binding, i.e., that unconscious phenomenon whereby by performing an action one perceives its effect earlier than when one is not the acting subject, was diminished by a reduction in voluntary control over an action. The participants were divided into three groups, with a first group (positive control) in which people voluntarily performed the action of pressing a button, a second group (no control) in which the button was pressed automatically, and a third group (negative control) in which the device gave the order to perform the action. In the second and third groups, intentional binding, an implicit indicator of agency, was reduced, but in subjects with psychosis-like traits, intentional binding was increased when they had no control and reduced in those with negative control. In the positive control group, subjects with positive social traits were found to have higher intentional binding, demonstrating that intentional binding and thus agency depend both on the environment (the possibility of acting) and on the characteristics of the social traits.

Another cognitive ability that is particularly deficient in UHR subjects is processing speed, whose deficit is considered by many authors to be the earliest neuropsychological alteration in the development of psychotic spectrum disorders, and one of the neurocognitive domains most implicated in schizophrenia. This is confirmed by the literature, which recognizes it as the first cognitive function to be impaired in the prodromal stages and in which it is also considered a marker of psychotic vulnerability [180]. Having established the presence of such alterations, some research on the high-risk state has investigated whether there are elements among the neurocognitive aspects that characterize it that could contribute to predicting the risk of transition to a full-blown psychotic state. The study conducted by Simon (2013) [181] reports, in addition to the neuropsychological description that characterizes these high-risk states just described, an in-depth meta-analysis that on the basis of seven studies aimed to identify cognitive characteristics that distinguish subjects at high risk of developing psychosis (UHR converters, *n* = 233) from those without such an outcome (UHR non-converters, *n* = 365). The hypothesis was that these differences could represent elements that are potentially predictive of the transition to psychosis. The results of this study show that UHR converter subjects have lower cognitive performance at baseline than controls, with greater impairments in general intelligence, visual verbal memory, working memory, and verbal fluency [182], supporting the idea that identifying cognitive phenotypes may improve our predictive ability. These aspects were then investigated in a subsequent meta-analysis by Bora and colleagues (2014) [183], which found that UHR subjects who developed psychosis had a significantly more severe cognitive impairment than the group in which psychosis had not developed, particularly affecting visual memory, verbal fluency, and working memory. More recently, another meta-analysis by Hauser and colleagues (2017) [184] confirmed that clinically high-risk (CHR) subjects show a mild to moderate global deficit in neuropsychological performance, representing a transient intermediate stage between first-episode psychotic subjects and healthy controls. Some cognitive deficits present in prodromal syndromes that may have predictive value are those in the areas of (verbal) memory and executive function, working memory, verbal fluency, processing speed, and verbal IQ [185]. An improvement in the prediction of the transition to full-blown psychosis seems to result from the combined assessment of positive and negative symptoms with certain cognitive symptoms, including in particular processing speed, as opposed to other deficits such as those affecting general intelligence and sustained attention [183,186]. It is thus clear that neuropsychology, together with clinical elements, would contribute to the creation of integrated predictive models that are more sensitive to the psychotic shift [187].

Another element of particular interest is the temporal aspect of neurocognitive abnormalities in UHR patients, an issue that could be further explored through comparative studies between ARMS subjects and first-episode psychotic patients. If the neurocognitive impairment in the UHR subject appears similar, if less severe, than in subjects with a full-blown disorder, differences have been found between UHR converters and non-converters [181,187,188]: the latter reported stabilization or even improvement in cognitive functions over time in the areas of attention, processing speed, executive functions, fine motor skills, and visuospatial working memory, whereas UHR converters showed a worsening of pre-existing neurocognitive impairments. Further differences would also be evident between those at an earlier stage of risk and those at a later stage (typically with attenuated psychotic symptoms), with the latter having more pronounced neurocognitive deficits [180]. This suggests that the progression of cognitive deficits in the development of psychotic disorder is aggravated, and particularly so at the clinical onset of the illness.

To better understand the functional changes that characterize these risk and psychotic transition periods, we have used neuroimaging studies with Magnetic Resonance Imaging (MRI), which have revealed structural and functional abnormalities in adolescent and young CHR compared to risk controls, like those observed in schizophrenic patients [163]. Alterations in synaptogenesis and synaptic pruning during neurodevelopment lead to bilateral reductions in grey matter density in regions such as the hippocampus, anterior cingulate cortex, frontal, temporal, and parietal lobes [189,190]. Consistent with the cognitive deficits observed in CHR subjects in the domains of attention, processing speed, and executive functions and memory, a lower density of intraglionic grey matter was detected [191]. Thus, changes at the neuroanatomical level, as well as those at the functional level, are also associated with an increased likelihood of transition: the most predictive and, over time, proven markers of transition risk are faster and more pronounced changes in grey matter. Indeed, longitudinal studies themselves have highlighted a faster rate of grey matter decline in CHR converters than in non-converters [192,193].

The comparison between these two classes of CHR subjects revealed the normal acceleration of these cortical deterioration processes, which also occur normally during physiological ageing. Specifically, a more rapid deterioration was observed at the level of the right prefrontal cortex and, bilaterally, the insula and the inferior frontal temporal cortex, in line with the general reduction in cognitive abilities in converter subjects compared to non-risk subjects. Neuroanatomical measures have been shown to be reliable not only in discriminating between clinically at-risk and healthy subjects, but also in discriminating between early- and late-stage CHR patients [151,192,194]. In particular, the alterations found in the latter two classes with respect to controls mainly concern prefrontal and temporal cortex, parietal, and subcortical structures responsible for executive functions, working memory and motor control, whereas with respect to early-risk CHR, late-risk CHR show more extensive reductions, consistent with a more pronounced symptomatic picture. It is also interesting to note that in late-risk CHR there is a reduction in the grey matter of the anterior and posterior cingulate, a structure strongly implicated in mediating between cognitive and emotional stimuli [195].

These findings were also confirmed by a recent meta-analysis by Millman (2022) [196] and co-workers, which examined neuropsychological performance in individuals at clinical high risk (CHR) for psychosis compared with clinical controls (CC) with other psychopathology. The results suggest that although both groups showed neurocognitive impairment compared to healthy individuals, there was little difference between CHR and CC when the later transition to psychosis was not taken into account. However, the subgroup of CHR individuals who eventually developed psychosis (CHR-T) showed significantly greater and more consistent cognitive deficits than both CC participants and CHR individuals who did not transition to psychosis (CHR-NT). This suggests that severe neurocognitive impairment is more closely associated with the onset of psychosis rather than being a general marker of CHR status.

The assessment of neuropsychological functions is essential for understanding schizophrenia, as cognitive impairments are strongly related to functional skills and outcomes [167,168]. General intellectual ability is significantly impaired, with IQ deficits greater than those seen in composite neuropsychological scores [196,197]. Therefore, total IQ can serve as a descriptive measure of overall intellectual ability and provide interpretive context for other test results. In this regard, Blyler et al. developed a short form of the Wechsler Adult Intelligence Scale (WAIS)-III, which is highly predictive of full-scale IQ in individuals with schizophrenia [198]. The MATRICS Consensus Cognitive Battery (MCCB) was developed through an expert consensus to evaluate cognition-enhancing agents in schizophrenia [199,200]. It also provides a standardized assessment of core cognitive deficits, identifying seven cognitive domains: speed of processing, attention/vigilance, working memory, verbal learning, visual learning, reasoning and problem-solving (executive functions), and social cognition. The MCCB takes about 60 min to complete, has alternate forms, and has been empirically validated with normative data [201]. A shorter battery, the Brief Assessment of Cognition in Schizophrenia (BACS), evaluates four of the seven MCCB cognitive domains: executive functions, processing speed, verbal memory, and working memory. It takes approximately 30 min to administer and has been validated with alternate forms and normative data. Studies using the interview-based Schizophrenia Cognition Rating Scale (SCoRS) have demonstrated substantial cognitive impairment in subjects with schizophrenia relative to healthy controls [202]. SCoRS has several notable strengths, including its relatively short administration time of approximately 15 min per interview, its established connection to real-world functioning, strong test–retest reliability, and its correlations with at least some performance-based cognitive measures [202,203]. However, several challenges remain. Given that individuals with schizophrenia often struggle to accurately report their cognitive abilities and daily functioning, the validity of SCoRS and its correlation with performance-based cognitive measures may be influenced by the availability of an informant [204,205].

Two commonly used cognitive screening tools for adults are the Screen for Cognitive Impairment in Psychiatry [206] and the Montreal Cognitive Assessment [207]. Among individuals with psychiatric conditions, the SCIP is generally preferred due to its emphasis on the most relevant cognitive domains and its proven effectiveness and acceptability in populations with schizophrenia, bipolar disorder, depression, and ADHD [208,209]. Additionally, research has shown that the SCIP exhibits strong interrater and test–retest reliability, as well as solid convergent validity, sensitivity, and specificity [210,211]. Conversely, the MoCA is primarily used in older adults suspected of having cognitive impairment, which is often an early indicator of dementia [212]. A recent study that evaluated the reliability and validity of both tools in a sample of adults with psychotic disorders (average age of approximately 43 years) found that the SCIP was more effective in detecting cognitive impairment than the MoCA [213]. The results of this study revealed empirical findings of accuracy of the CogScreen classification will provide empirical evidence of the validity and reliability of two cognitive screening instruments (e.g., SCIP and MoCA) in a youth population compared to a gold standard neuropsychological assessment battery [214].

Despite the extensive research on cognitive assessment in schizophrenia, a significant limitation is the lack of standardized test batteries, specifically designed to identify early signs of psychosis or prodromal symptoms. The existing literature, while vast, predominantly focuses on cognitive evaluation in individuals with established psychotic symptoms rather than on early detection. Current assessments, such as the MCCB, BACS, and SCoRS, are effective in characterizing cognitive deficits but may not be sensitive enough to detect subtle impairments preceding the onset of full-blown psychosis. Future challenges will involve developing and standardizing cognitive tools with high sensitivity to early deficits, enabling more precise early identification and intervention strategies.

## 7. The Relevance of Early Diagnosis and Aberrant Salience Assessment in the Prevention of Psychosis

Since psychosis is a severe psychiatric disorder characterized by a loss of contact with reality, which may manifest through delusions, hallucinations, and disorganized thinking, understanding the prodromal symptoms, particularly aberrant salience, is crucial for early diagnosis and effective intervention. The early diagnosis of psychosis constitutes a pivotal component of clinical management, as it has been demonstrated to be associated with enhanced long-term prognoses [215]. Aberrant salience, defined as the perception of everyday events or stimuli as excessively significant or relevant, frequently emerges as one of the initial symptoms experienced by patients [216]. Its timely identification is therefore of great importance in order to prevent the onset of full-blown psychotic episodes. Assessment tools such as the Aberrant Salience Inventory (ASI) and the Scale of Prodromal Symptoms (SOPS) have been developed to systematically measure these symptoms and identify patients at risk [217,218].

Recent findings from dynamic network neuroscience have shown that aberrant salience is linked to dysfunctional interactions among large-scale brain networks. Specifically, a triple-network model involving the salience network (SN), the frontoparietal network (FPN), and the default mode network (DMN) has been proposed. This model suggests that impaired coordination among these networks—particularly the SN’s role in identifying and responding to salient stimuli—may underlie psychotic symptomatology. In patients with schizophrenia, the dynamic interactions between these networks are less persistent, more variable, and reduced in strength compared to healthy controls. These alterations are associated with positive symptoms, indicating a central role of the SN in modulating salience and regulating cross-network communication [219].

Additionally, individuals at clinical high risk for psychosis show aberrant connectivity between the SN and DMN, notably a loss of the typical anti-correlated pattern between them. This disruption correlates with cognitive decline and reality distortion symptoms, suggesting SN dysfunction as a predictor of adverse cognitive and thought outcomes [220].

Importantly, psychosis-proneness is increasingly understood as a psychological dimension that extends into the general population. Psychotic-like traits—such as the tendency to assign meaning to irrelevant stimuli—can be observed in healthy individuals and are linked to individual differences in brain connectivity and dopaminergic activity. For example, ultra-high-risk individuals tend to attribute excessive salience to irrelevant stimulus features and show striatal responses related to delusion-like symptom severity, implying that salience dysregulation may be a transdiagnostic mechanism spanning health and illness [221].

Moreover, several factors can exacerbate psychosis-proneness, including reduced sense of agency, interaction with intrusive technologies, and adverse or ambiguous environments. A diminished sense of agency—the experience of not being the cause of one’s own actions—has been associated with hallucinations and delusions. Altered predictive processing underlying action and control may play a key role in these experiences. Furthermore, loss of voluntary control in task performance has been shown to decrease implicit indicators of agency, suggesting that impaired agency may shape self-perception and increase vulnerability to psychotic phenomena [222].

Various tools have been developed to assess high-risk mental states, often used in synergy. Among the most well-known for identifying individuals in the prodromal phase are the Comprehensive Assessment of At-Risk Mental State (CAARMS), the Structured Interview for Prodromal Syndromes (SIPS), which includes the SOPS, and the Schizophrenia Proneness Instrument in its adult (SPI-A) and child-adolescent versions (SPI-CY). These interviews, used for both clinical and research purposes, require specific training, involve long administration times, and are often conducted in Early Intervention centers. Additionally, more accessible screening tools have been developed to improve patient identification, including the PRIME screen, the Youth Psychosis at Risk Questionnaire-Brief, the Prodromal Questionnaire (PQ), and the Checklist ERIraos [223,224,225,226].

The Aberrant Salience Inventory (ASI) has been developed to measure the concept of “aberrant salience”, which is essential for understanding psychotic processes. The ASI comprises questions that explore various dimensions of subjective experience, such as attention to irrelevant details and the perception of hidden connections between events. Participants respond to these questions on a Likert scale, thereby quantifying the level of perceived aberrant salience. The primary objective of the ASI is to identify individuals with a predisposition to psychotic thinking, thus enabling early interventions. The ASI has been shown to be a valuable research and clinical instrument [227]. Recent studies have demonstrated that the implementation of screening for aberrant salience in high-risk groups, such as young adults with a family history of psychosis, can result in targeted early interventions, including cognitive-behavioral therapy and pharmacological monitoring, with significant effect. This has been demonstrated by a significant reduction in the incidence of psychosis in high-risk groups, as evidenced by recent studies [216].

The Checklist ERIraos, developed by Maurer et al. (2006), combines nonspecific distress indicators, often present in the prodromal phases of psychosis, such as social withdrawal, suspiciousness, depression, lack of energy, and nervousness, with symptoms that are more psychopathologically characteristic of psychosis, such as derealization, persecutory ideation, ideas of reference, and hallucinatory phenomena [228,229]. It consists of 17 items inspired by the Interview for the Retrospective Assessment of Schizophrenia (IRAOS), basic symptoms, CAARMS, and SIPS. It evaluates observed changes in the subject compared to the previous period and documents the presence of a family history of schizophrenia and/or psychiatric disorders, as well as obstetric and perinatal complications. This tool is suitable not only for use in psychiatric services but also in General Medicine, Pediatrics, Family Planning Clinics, and Emergency Departments. A score of 12 or higher indicates the need for further diagnostic evaluation at a specialized center. Since it is a semi-structured interview, its reliability is ensured by the examiner’s specific training, ability to detect nuances and hesitations in often ambiguous and uncertain responses, deep knowledge of the psychopathological areas examined, and the patient’s understanding of the symptoms [229].

The Prodromal Questionnaire initially consisted of 92 items and was later significantly reduced in the Brief version (PQ-B) and in the 16-item version (PQ-16), which is also available for children. The PQ-16, most commonly used for screening purposes, includes nine items for perceptual anomalies and subthreshold hallucinations, five items regarding unusual thought content, delusional ideas, and paranoia, and two items specifically for negative symptoms. However, none of these versions consider family history of psychotic disorders or the presence of schizotypal personality, making their integration necessary when needed. The subject must estimate both the presence of the symptom and the self-perceived level of distress. The cut-off for help-seeking individuals is a score ≥ 6 [225].

The Schizophrenia Proneness Instrument Adult Version (SPI-A) and Child-Youth (SPI-CY), stem from the concept of basic symptoms, first described by Gerard Huber (1983) [76]. They are semi-structured interviews that assess the type, frequency, and severity of two groups of basic symptoms [230]: cognitive-perceptual symptoms (COPER) (10 items, including intrusive thoughts, thought pressure, thought blocking, receptive language disturbances, reduced ability to distinguish between ideas and perceptions or between fantasies and memories, ideas of reference, derealization, visual perception disturbances, and auditory perception disturbances) and cognitive disturbances (9 items, including inability to divide attention, intrusive thoughts, thought pressure, thought flow blockage, receptive language disturbances, expressive language disturbances, inconsistent ideas of reference, disturbances in symbolic interpretation, and a tendency to focus excessively on small details).

Like the previous tools, the Comprehensive Assessment of At-Risk Mental State (CAARMS) is a semi-structured interview developed by the Australian group at the PACE Clinic in Melbourne [88]. It identifies help-seeking individuals who present signs and symptoms consistent with ultra-high-risk (UHR) criteria, confirms or excludes a psychotic onset, and monitors symptoms over time. This tool is highly versatile as it measures the frequency, intensity, duration, and distress levels of symptoms, while also assessing substance use or abuse and determining the subject’s UHR group classification [231]. To enhance sensitivity and specificity, CAARMS is supplemented with the Social and Occupational Functioning Assessment to estimate the patient’s functional decline [232]. There are two versions of this scale. The full version, mainly used for research, is organized into seven domains (positive symptoms, cognitive changes (e.g., memory difficulties), negative symptoms (e.g., alogia, apathy, anhedonia), behavioral changes (e.g., social withdrawal, aggressive or risky behaviors), physical/motor changes (e.g., disorganized movement, mannerisms, bodily change sensations), emotional disturbances, and general psychopathology (e.g., obsessive thoughts, mood disorders, anxiety). The brief version, primarily used in clinical settings for easier execution [233].

The Structured Interview for Psychosis Risk Syndromes and Scale of Prodromal Symptoms (SIPS/SOPS) is one of the most important psychiatric assessment scales [164]. It excludes the presence of past or current psychotic disorders and identifies the three UHR subgroups of psychosis risk syndromes, estimating symptom severity: brief intermittent psychotic syndrome, attenuated positive symptom syndrome, and genetic risk and deterioration syndrome. Risk is assessed comprehensively by evaluating symptom intensity, frequency, functional deterioration, and genetic risk. This tool is structured into multiple sections, including the Scale of Psychosis-Risk Symptoms (SOPS) (19 items), the Global Assessment of Functioning (GAF), an assessment for schizotypal personality disorder, criteria for prodromal syndrome (COPS) and full-blown psychosis (POPS), a psychiatric history assessment.

To provide a comparative overview, Table 3 summarizes the major psychometric tools used for UHR detection.

Beyond early identification, structured psychometric profiling allows tailoring preventive interventions according to specific cognitive and clinical patterns:

- Aberrant salience predominance (ASI, CAARMS positive symptom domains): Individuals showing elevated aberrant salience or early positive symptoms can benefit from Cognitive-Behavioral Therapy for Psychosis Prevention (CBTp) [216,227].
- Basic cognitive disturbances (SPI-A, SPI-CY profiles): Symptoms such as thought interference, receptive language disturbances, and derealization indicate vulnerability to psychosis. Metacognitive Training (MCT) and Cognitive Remediation Therapy can enhance cognitive flexibility and insight, reducing risk [78].
- Functional deterioration and psychosocial decline (SIPS/SOPS, CAARMS functioning domains): Early identification of social withdrawal, academic failure, and occupational decline suggests integrative approaches combining psychoeducation, social skills training, and vocational support [231,232].
- Distressful subthreshold psychotic symptoms (PQ-16, Checklist ERIraos): These indicate the need for low-threshold cognitive-behavioral interventions and active symptom monitoring. In cases of progression, low-dose pharmacological treatments may be considered [223,224,225].
- Genetic risk with symptom exacerbation (SIPS genetic risk criteria): Strong familial predisposition combined with functional deterioration suggests integrated pharmacological and psychosocial preventive strategies [164].

Thus, assessment tools are not merely diagnostic instruments; they actively inform clinical pathways, allowing the personalization of preventive strategies according to symptom dimensions and risk profiles. Such a precision psychiatry approach can significantly improve outcomes by intervening early, when therapeutic leverage is highest. Moreover, emerging research suggests that the combination of psychometric profiling with biomarkers of brain network instability, such as default mode network alterations [162], could further refine individualized prevention strategies.

## 8. Therapeutic Approaches and Targeted Intervention Strategies in Psychosis Prevention

The therapeutic approach to psychosis should be integrated and multidisciplinary, combining pharmacological and non-pharmacological strategies. The use of antipsychotics, particularly second-generation ones, has proven effective in treating psychotic symptoms and improving overall functioning in patients.

Antipsychotics are the primary class of medications used to treat aberrant salience. These medications function by obstructing dopamine D2 receptors within the brain, thereby diminishing the heightened dopaminergic activity that can result in the misattribution of significance to irrelevant stimuli. This process assists in the reduction of positive psychotic symptoms, such as hallucinations and delusions. Typical antipsychotics, including haloperidol, have proven efficacious in the management of aberrant salience; however, they can also lead to substantial side effects, including extrapyramidal symptoms and tardive dyskinesia. Antipsychotics such as clozapine, olanzapine, and risperidone are frequently favored due to their more favorable side-effect profile, as they not only block dopaminergic receptors but also act on other neurotransmitter systems, such as the serotoninergic system, further improving the control of psychotic symptoms [234,235]. However, it is crucial to closely monitor medication administration to avoid side effects, such as the risk of metabolic syndrome associated with prolonged use of antipsychotics [236].

Cognitive and emotional dysregulation represents a crucial aspect of aberrant salience and is one of the primary challenges for patients experiencing psychotic symptoms or at risk of developing them. Emotional dysregulation refers to the difficulty in regulating emotions in a manner consistent with the situation. In psychotic disorders, this difficulty can lead to exaggerated responses to everyday stimuli, where the perception of neutral or insignificant stimuli as threatening or significant becomes particularly intense. This phenomenon is characterized by symptoms such as anxiety, paranoia, and a distorted view of reality, which perpetuate the cycle of stress and psychological distress [229]. Treatment of emotional dysregulation in the attribution of salience primarily focuses on improving the patient’s ability to identify, express, and regulate their emotions more functionally [230]. Managing emotional dysregulation is especially important as strongly disorganized emotions can contribute to the intensification of psychotic symptoms and difficulty recognizing and separating relevant stimuli from irrelevant [231]. Several therapeutic approaches have proven effective in treating this aspect in patients with psychotic disorders, such as cognitive-behavioral therapy (CBT) and mindfulness [230].

CBT assists patients in identifying misguided thought patterns and erroneous beliefs that precipitate inappropriate emotional responses, such as fear and paranoia. Utilizing techniques like cognitive restructuring, patients learn to recognize and rectify the thoughts that amplify the significance attributed to quotidian stimuli. The CBT approach has been shown to reduce the intensity of aberrant salience and to improve emotional regulation, allowing patients to respond in a more adaptive and proportionate manner to environmental stimuli [232]. A fundamental component of CBT for emotional dysregulation is psychoeducation, which equips patients with the tools to comprehend and manage their emotional states. This approach has been found to be particularly beneficial in cases where emotional difficulty is linked to traumatic experiences or stressful events that amplify the perception of danger in social or interpersonal contexts [233,234]. Mindfulness techniques represent another approach that has proven effective in treating emotional dysregulation in patients with aberrant salience. Mindfulness focuses on increasing awareness of the present moment, encouraging patients to observe their thoughts and emotions without judgment. This practice has been shown to be particularly helpful in reducing exaggerated emotional reactivity, promoting better emotional regulation through non-reactive awareness and self-compassion [235,236]. Mindfulness-Based Therapy (MBT), which integrates mindfulness with conventional therapeutic techniques, has been demonstrated to enhance emotional well-being in patients diagnosed with psychosis. Specifically, MBT assists patients in discerning intrusive thoughts from authentic threats, thereby diminishing avoidance behaviors and self-criticism. This approach has been linked with enhanced psychological flexibility, empowering patients to manage their internal experiences without succumbing to overwhelming distress [235,236].

The treatment of emotional dysregulation in aberrant salience requires a complex approach that integrates both psychotherapeutic and pharmacological interventions. Cognitive-behavioral therapy, mindfulness-based interventions, and dialectical behavior therapy provide a solid foundation on which to build, helping patients to improve their emotional regulation and reduce the overvaluation of environmental stimuli. The integration of these techniques with pharmacological support, when needed, can enable more effective symptom management and enhance the patient’s overall well-being. Timely and personalized treatment is essential to prevent psychotic deterioration and improve the quality of life in patients at risk of psychosis.

Research is ongoing, with studies exploring new potential treatments to modulate aberrant salience without the typical side effects of antipsychotics. Recent studies have highlighted the importance of metabotropic glutamate receptors (mGlu) in schizophrenia. In particular, mGlu5 receptors have been identified as promising therapeutic targets. Positive allosteric modulators of these receptors can increase the glutamate response, affecting synaptic transmission and potentially improving psychotic symptoms. Recent studies suggest that glutamate’s influence could offer new therapeutic possibilities [237,238]. Other studies have been conducted on N-methyl-D-aspartate (NMDA) receptor dysfunction. NMDA receptor hypofunction has been associated with the pathophysiology of schizophrenia [239]. Modulation of this system through specific drugs could contribute to the treatment of aberrant salience and psychotic symptoms. According to a study by Javitt et al. (2023), NMDA receptor antagonism can reproduce psychotic symptoms similar to those of schizophrenia, confirming the importance of the glutamatergic system in the disorder’s pathogenesis [240].

The interaction between dopaminergic and serotonergic systems is complex and plays a crucial role in schizophrenia. Alterations in the serotoninergic system have been linked to psychiatric disorders such as depression, anxiety, and schizophrenia. Modulating these pathways could further improve therapeutic outcomes, as serotoninergic receptors influence dopamine release and vice versa, highlighting the importance of this link in schizophrenia treatment [105]. More recent studies, such as that by Kim S (2021), show that modulation of the 5-HT1A serotoninergic receptors may lead to improvements in psychotic symptoms and salience regulation [241].

## 9. Conclusions and Future Directions

Aberrant salience is increasingly recognized as a key neurocognitive mechanism in the development of psychosis. It represents a fundamental alteration in the way individuals process environmental stimuli, leading to the misattribution of significance to otherwise neutral cues. Research on young individuals at ultra-high risk (UHR) for psychosis has demonstrated that aberrant salience may serve as an early biomarker for psychotic transition. By identifying and targeting these disruptions in salience attribution, researchers can refine predictive models of psychosis and improve early detection efforts. Given its role in the prodromal phase of psychosis, aberrant salience should be a primary focus in neurobiological and clinical studies aimed at understanding the mechanisms underlying psychotic disorders.

This manuscript provides ample groundwork for a structured comparison of neurobiological, cognitive, and clinical markers of aberrant salience, and it lends itself well to proposing a conceptual model to inform early intervention strategies in ultra-high-risk (UHR) populations. A systematic integration of neurobiological, cognitive, and clinical markers of aberrant salience offers a promising framework for improving early detection and intervention in individuals at UHR for psychosis. Neurobiologically, aberrant salience is underpinned by dysregulations in dopaminergic, glutamatergic, and GABAergic signaling, particularly within the mesolimbic pathway, prefrontal cortex, and salience-related structures such as the anterior cingulate cortex, insula, and striatum. These abnormalities result in altered prediction error signaling and impaired reality testing, as articulated in the i-RISA and Bayesian inference models. At the cognitive level, deficits in attention, working memory, and processing speed (alongside impaired social cognition) reflect disruptions in higher-order executive control and are predictive of transition to psychosis in UHR converters. Clinically, these mechanisms manifest through basic symptoms (BS), Attenuated Psychotic Symptoms (APS), and Brief Limited Intermittent Psychotic Symptoms (BLIPS), which can be systematically captured using tools such as the CAARMS, SPI-A, and the Aberrant Salience Inventory (ASI). Together, these markers support a dimensional, stage-based model of psychosis vulnerability that situates aberrant salience as a transdiagnostic mechanism and a state-sensitive biomarker. This integrative model not only advances theoretical understanding of psychosis onset but also provides a pragmatic scaffold for tailoring multimodal, early intervention strategies—including cognitive remediation, antipsychotic prophylaxis, and psychotherapeutic modulation of salience attribution—in youth at elevated clinical risk (Table 4).

Despite growing evidence, several questions remain regarding the trajectory of aberrant salience in UHR populations. Longitudinal studies are necessary to determine whether aberrant salience remains a stable trait or fluctuates depending on stressors, neurochemical imbalances, and environmental influences. Additionally, intervention strategies need to be further explored, particularly in non-pharmacological domains such as cognitive remediation, mindfulness-based interventions, and targeted psychotherapy approaches aimed at improving reality testing and emotional regulation. Pharmacological interventions addressing dopamine dysregulation should also be further refined, with an emphasis on personalized treatment strategies for individuals exhibiting heightened salience dysregulation.

Integrating aberrant salience assessments into routine clinical evaluations of UHR individuals may enhance early intervention efforts. Standardized tools should be widely implemented to identify at-risk individuals. Furthermore, clinicians should adopt a multimodal approach that combines neuroimaging, cognitive testing, and clinical assessments to improve diagnostic accuracy and tailor interventions. For example, early intervention centers should incorporate psychoeducational strategies that help individuals manage salience disturbances before they evolve into full-blown psychotic symptoms.

Future research and clinical advancements must align to refine risk prediction models, optimize interventions, and ultimately prevent the onset of psychosis in vulnerable individuals. By addressing aberrant salience at its roots, we can move toward a more targeted and effective approach in psychosis prevention and early treatment.

## Figures and Tables

**Table 1:** Perceptual impairments and cognitive disturbances in ultra-high-risk (UHR) population.

Perceptual Impairments (Cognitive Perceptive, COPER) and Cognitive Disturbances (COGDIS)
- Thought interference: subjective experience related to the presence of intrusive thoughts that appear to the young person as completely meaningless and hinder concentration
- Persistent thinking: obsessive repetition of meaningless thoughts or mental images
- Thought pressure: reported chaos of unrelated thoughts
- Mental blocks with or without intrusions of a new thought: a sudden loss of the thread or train of thought
- Alteration of receptive language: paralysis in immediate comprehension of simple words and sentences, read or heard, which may result in abandoning reading or avoiding conversations
- Expressive language impairment: problems in producing appropriate words, sometimes subjective experience of loss of active vocabulary
- Abstract thinking disorders: difficulty for the patient to explain sayings or idioms
- Difficulty with distributed attention: difficulty in dividing attention between tasks that are not given simultaneously and do not usually require a shift in attention
- Decreased ability to discriminate between perception and ideas, good memories and fantasies
- Unstable idea of reference with intuition
- Derealization and depersonalization
- Visual or auditory perceptual disturbances with preserved insight, e.g., hypersensitivity to light, distorted vision, hypersensitivity to sounds

**Table 2:** The Clinical Stage Model.

Stage	Psychosis	Treatment
**0**	No symptoms Genetic risk	Promote knowledge and dissemination of accurate mental health information and drug use prevention. Educational interventions aimed at families.
**1a**	Non-specific symptoms, basic cognitive symptoms	Interventions similar to the previous stage, with the addition of psychoeducational interventions and cognitive-behavioral therapy aimed at reducing distress caused by the onset of symptoms
**1b**	Attenuated psychotic symptoms (APS)	Interventions similar to the previous stage, with the addition of individual cognitive-behavioral therapy and possible pharmacological treatment for comorbid anxiety and depression symptoms
**1c**	Brief limited intermittent psychotic symptoms (BLIPS)	Interventions aimed at the remission of psychotic symptoms
**2**	First psychotic episode	Early intervention for the first psychotic episode, as in stage 2b, with greater emphasis on pharmacological treatments and social support for role maintenance. Relapse prevention
**3a**	Incomplete remission	Interventions from the previous stage, with more emphasis on pharmacological therapy and psychosocial support strategies to facilitate symptom remission
**3b**	Relapse	Interventions similar to the previous stage, with more attention to relapse prevention strategies
**3c**	Recurring relapses with clinical deterioration	Interventions similar to stage 3b, with greater focus on long-term stabilization
**4**	Severe and persistent disorder	Interventions similar to the previous stage with possible introduction of clozapine. Encourage social participation to address ongoing disability

**Table 3:** Major psychometric tools used for UHR detection.

Instrument	Domains Covered	Sensitivity/Specificity	Clinical Applicability
CAARMS	Positive symptoms, cognitive changes, negative symptoms, behavioral and emotional disturbances	High/Moderate	Specialized centers (early intervention, research)
SIPS/SOPS	Psychosis-risk symptoms, functional deterioration, genetic vulnerability	High/High	Specialized centers, longitudinal monitoring
SPI-A/SPI-CY	Basic cognitive-perceptual anomalies	High/Moderate	Early detection in youth and adults
ASI	Aberrant salience attribution (subjective anomalies)	Moderate/Moderate	High-risk group screening, research contexts
PQ-16	Perceptual anomalies, unusual thoughts, negative symptoms	Moderate/Low	Rapid community screening, primary care
Checklist ERIraos	Early distress and psychotic symptoms, family risk factors	Moderate/Moderate	Broad screening in general practice

**Table 4:** Markers of aberrant salience in ultra-high-risk populations.

Domain	Marker/Feature	Implication for Aberrant Salience	Assessment Tools	Clinical Implications
Neurobiological	Dopaminergic dysregulation (mesolimbic pathway)	Hyperdopaminergia leads to inappropriate salience attribution	PET, fMRI, DTI imaging	Supports early use of dopamine-modulating agents; risk stratification via PET imaging.
Neurobiological	Glutamatergic and GABAergic imbalance	Loss of inhibitory tone affects salience processing and prediction error	MR spectroscopy, post-mortem studies	Indicates potential for glutamate-targeted interventions (e.g., NMDA enhancers).
Neurobiological	Salience network dysfunction (ACC, insula, striatum)	Disrupted connectivity leads to impaired filtering and evaluation of stimuli	Resting-state fMRI, DTI connectivity studies	Supports use of network-targeted neurofeedback or neuromodulation strategies.
Cognitive	Deficits in attention	Difficulty in filtering relevant from irrelevant stimuli	Neuropsychological batteries (e.g., MATRICS, BACS)	Recommends early cognitive training and attention-enhancement therapies.
Cognitive	Impaired working memory	Poor integration of information increases cognitive noise	Working memory tests (e.g., WAIS subtests)	Justifies inclusion of working memory training in early intervention programs.
Cognitive	Slowed processing speed	Reduces capacity to adjust to complex environments, enhancing salience misattribution	Processing speed tasks (e.g., Trail Making Test)	Highlights need for strategies enhancing processing speed to reduce overload.
Cognitive	Social cognition deficits	Impaired understanding of social cues fosters delusional interpretations	Theory of mind and facial emotion recognition tasks	Indicates benefits from social cognition-focused therapies (e.g., ToM training).
Clinical	Basic Symptoms (BS)	Subjective anomalies linked to emerging misattribution of significance	SPI-A/SPI-CY (Schizophrenia Proneness Instruments)	Alerts clinicians to subtle self-disturbances; use of BS-based screening tools.
Clinical	Attenuated Psychotic Symptoms (APS)	Emergence of mild, persistent misattributions of meaning	CAARMS (Comprehensive Assessment of At-Risk Mental States)	Supports continuous symptom monitoring and use of CBT to delay progression.
Clinical	Brief Limited Intermittent Psychotic Symptoms (BLIPS)	Short-lived but fully formed salience-related perceptual disturbances	SIPS/SOPS, clinical interviews	Justifies close follow-up and rapid intervention strategies post-BLIPS episodes.

## Data Availability

No new data were created for this study.
